# Relationship between composite dietary antioxidants index and growth indicators in children aged 3–12 years: results from two observational studies

**DOI:** 10.3389/fnut.2025.1551754

**Published:** 2025-03-20

**Authors:** Rui Wen, Huanting Pei, Jingyi Ren, Siqi Zhu, Simeng Qiao, Pui Yee Tan, Yunyun Gong, Min Yang, Junsheng Huo, Gangqiang Ding, Yuxia Ma

**Affiliations:** ^1^Department of Nutrition and Food Hygiene, School of Public Health, Hebei Medical University, Hebei Key Laboratory of Environment and Human Health, Shijiazhuang, China; ^2^School of Food Science and Nutrition, Faculty of Environment, University of Leeds, Leeds, United Kingdom; ^3^School of Public Health, and Center of Clinical Big Data and Analytics of The Second Affiliated Hospital, Zhejiang University School of Medicine, Hangzhou, Zhejiang, China; ^4^National Institute for Nutrition and Health, Chinese Center for Disease Control and Prevention, Beijing, China

**Keywords:** dietary antioxidants, composite dietary antioxidant index, children growth, cross-sectional study, Chinese children

## Abstract

**Background:**

The intake of nutrients with antioxidant properties is closely related to numerous health outcomes. However, the evidence regarding the effects of antioxidant nutrient intake on children's growth indicators is still lacking. This study aimed to investigate the relationship between the Composite Dietary Antioxidant Index (CDAI) and child growth indicators in two cohorts.

**Methods:**

This study utilized data from 1,064 participants in the Children's Cohort Study on Micronutrient Deficits and Malnutrition (CCSMDM) 2023 database and 2,404 participants from the National Health and Nutrition Examination Survey (NHANES) 2003–2020 database for cross-sectional analyses, with participants aged 3–12 years. The association between CDAI and growth indicators was analyzed using multinomial logistics regression. And we also performed subgroup analyses to determine whether there were differences in gender and explored the dose-response relationship by fitting a restricted cubic spline.

**Results:**

After adjusting for potential confounders, CDAI was positively associated with children growth indicators (height: CCSMDM: OR =1.21, 1.04~1.43, *p* = 0.017; NHANES: OR = 1.11, 1.04~1.18, *p* = 0.001; weight: CCSMDM: OR =1.27, 1.09~1.52, *p* = 0.004; NHANES: OR = 1.12, 1.05~1.19, *p* < 0.001). Our study also found that there was a significant correlation between antioxidant nutrients (vitamin A, vitamin E, zinc, and magnesium) and height. And selenium, magnesium, and BMI were in close contact. Subgroup analysis found that CDAI had a higher positive association with height in male children.

**Conclusions:**

Our study revealed the benefits of dietary antioxidant nutrients for children growth indicators. These results suggested that a higher level of dietary antioxidant nutrients may help to promote children growth indicators. It is recommended to consume a combination of multiple antioxidants, as their interactions may offer potential benefits. However, further research is needed to explore the underlying mechanisms of the synergistic effects of antioxidants on children's growth and development.

## 1 Introduction

According to statistics, 155 million children under 5 years of age were stunted globally (1). And over 340 million children and adolescents were overweight or obese, which poses significant long-term health risks (2, 3). At present, children's growth indicators have garnered immense attention from the public alike. A report by the United Nations Children's Fund (UNICEF) pointed out that providing better nutrition, play, and early learning experiences in the critical years of life is crucial for Children's growth and development. The growth and development of children are complex processes that are influenced by various factors, including environmental factors, medical diseases, and nutritional status, etc. (4, 5). Studies have demonstrated that long-term accumulation of toxins in air and soil may adversely affect children's physical development and contribute to developmental delays, while malnutrition has been linked to increased child mortality and impairment of neurocognitive functioning (6). Out of these factors, reasonable dietary structure is particularly crucial to the growth and health of children. Imbalanced intake of nutrients can affect cognitive level, growth and development, and learning ability in children (7–9). The World Health Organization (WHO) also emphasizes the importance of children's dietary diversity and nutritional balance (10, 11). A survey showed that long term micronutrient deficiencies can also lead to the occurrence of some diseases, such as iron deficiency anemia and stunted growth due to zinc deficiency (12, 13). Therefore, a balanced intake of energy and macro- and micronutrients is important during this sensitive period of growth and development. By promoting healthy eating habits and providing proper nutritional support, we can help children reach their full growth potential and pave the way for a healthier, more prosperous future.

Oxidative stress (OS) is defined as an imbalance between oxidants (such as reactive oxygen species and nitrogen species) and antioxidants (such as antioxidant enzymes and superoxide dismutases), which disrupts redox signaling and control and/or result in molecular damage (14). This imbalance can trigger inflammatory responses, which further combine oxidative stress and inflammatory reactions to produce reactive oxygen species, leading to chronic diseases (15). Previous studies have found that malnutrition is closely associated with OS (9). Chronic OS could lead to damage of macromolecules (lipids, carbohydrates, proteins, and nucleic acids), cell degeneration, and death (16). Therefore, Such molecular and cellular damage is not conducive to children growth indicators, including linear growth, weight gain, and cognitive function. Recent studies have confirmed that children's dietary intake of antioxidant compounds possesses notable antioxidant potential, which may help mitigate the onset of OS (17, 18). Dietary micronutrients, including vitamins C and E, selenium, and zinc, play a crucial role in mitigating OS due to their antioxidant properties (19). A cross-sectional study conducted by Chiplonkar et al. (20) showed that dietary micronutrient deficiencies (i.e., calcium, iron, zinc, and β-carotene) were negatively correlated with linear growth. Another cohort study showed that gestational deficiency of the referred minerals seriously affected children growth indicators (21). The above evidence suggests that sufficient dietary intake of antioxidant nutrients is essential for children growth indicators.

Composite Dietary Antioxidant Index (CDAI) was first proposed by Wright et al. and is used to assess the total antioxidant capacity of an individual's diet (22). As an effective, flexible, and comprehensive nutritional assessment tool, CDAI is constructed according to their aggregate anti-inflammatory impact and has been proven to be closely related to the occurrence of a variety of diseases (23). However, there is no research that explores the relationship between CDAI and children growth indicators. In this study, our aim was to investigate the link between CDAI and children's growth indicators in two cohorts to examine the potential impact of dietary interventions on children's growth and development.

## 2 Methods

### 2.1 Study design and population

This study used Children cohort study on micronutrient deficiencies and malnutrition (CCSMDM) 2023 database and National Health and Nutrition Examination Survey (NHANES) 2003–2020 databases for cross-sectional analyses, with participants aged 3–12 years. The CCSMDM cross-sectional study was conducted in China to investigate the dual burden of malnutrition among Chinese children. The participants were selected using multi-stage stratified random sampling in four cities across the country to investigate their basic information, dietary data, and physical measurement data. The project was approved by the China Institute of Human Genetic Resources for International Cooperation and each participants agreed to sign an informed consent form.

The NHANES database was conducted by the Centers for Disease Control and Prevention (CDC) that focused on health and nutrition status of non-institutionalized children and adults in the United States, employing a complex multi-stage sampling design, and with data release in 2-year cycles (24). The research was approved by CDC National Center for Health Statistics Institutional Review Board, and written informed consent was obtained from all the participants and/or their parents.

**Figure 1:**
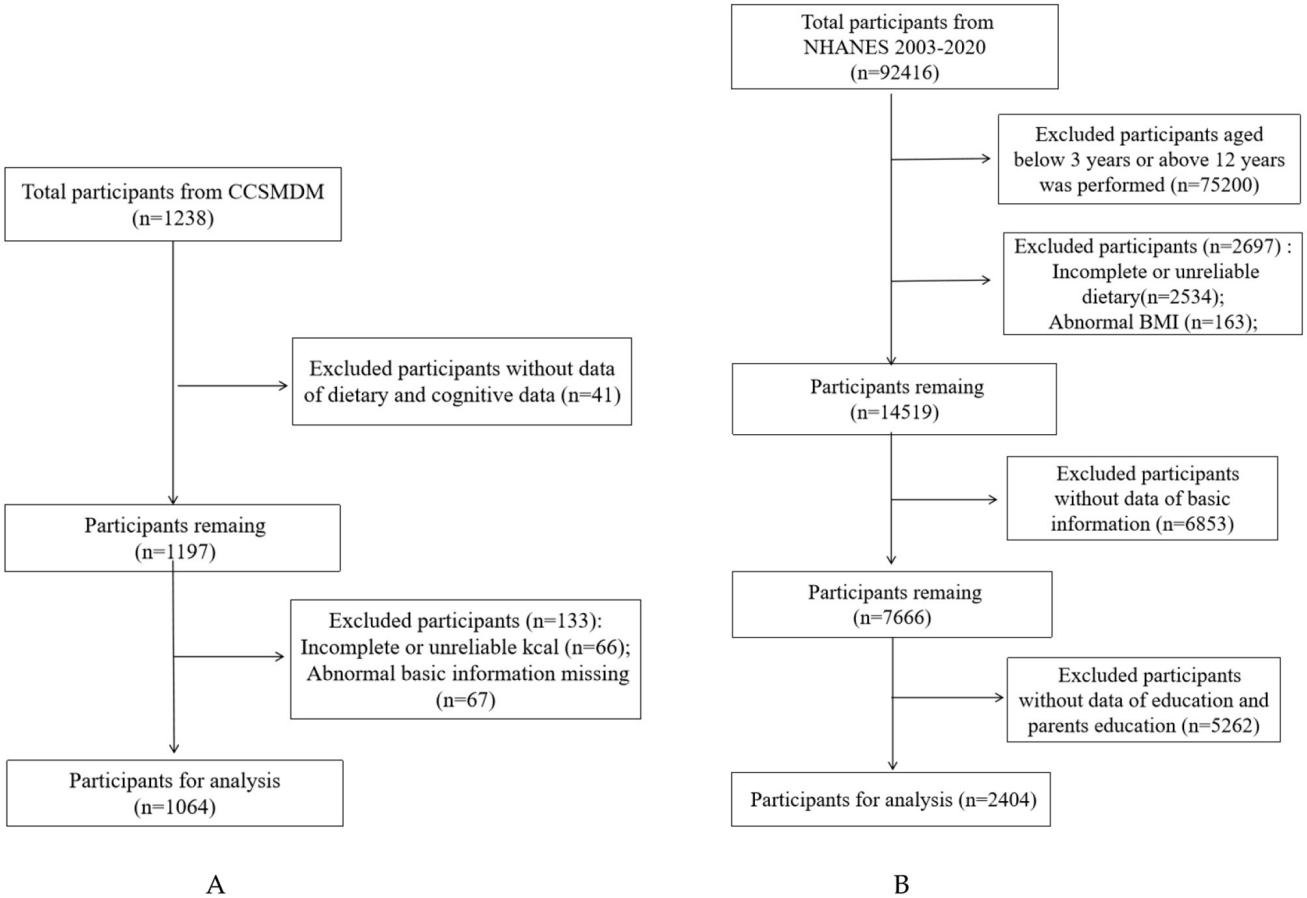
Flow chart of sample selection from the two cross-sectional studies. The **(A)** is from CCSMDM 2023; The **(B)** is sourced from NHANES 2003–2020.

We enrolled a total of 1,238 participants 3–12 years of age in 2023 CCSMDM and 92,416 participants 3–12 years of age in 2003–2020 NHANES, applying specific exclusion criteria. The exclusion criteria for this study were: (1) lacking basic information of participants, such as age, gender, race, education level; (2) missing dietary data and body measurement data; (3) extreme energy data (total energy intake <500 or >4,500 kcal/day). Ultimately, this study included 1,064 participants in 2023 CCSMDM and 2,404 participants in 2003–2020 NHANES (Figure 1).

### 2.2 Dietary assessment

In this study, dietary intake was assessed using two methods: the 24-h weighing method and the 24-h dietary recall method.

In the CCSMDM database, a 24-h dietary weighing method was used to assess dietary intake, which refers to recording the weight of all intake food and beverages for three consecutive days (two weekdays and one weekend day) through the weighing method. Then, we used the average 3-day dietary intake data combined with the Chinese Food Ingredient Table to calculate the daily average intake (25).

NHANES nutritional data consists of twice 24-h dietary reviews, recording the nutritional content food and beverages (26). The first dietary recall was collected in person; the second dietary interview proceeded 3–10 days by telephone. Participants were asked to recall details of all 24-h diets. After that the average daily intake was calculated based on the average of a 2-day dietary review, combined with the Food and Nutrient Database for Dietary Studies (FNDDS) of USDA data (27–29).

The CDAI is a nutritional indicator of validity and reliability, used to evaluate the total antioxidant properties in the diet, which is the total score of multiple dietary antioxidants (zinc, magnesium, selenium, vitamins A, vitamins C, and vitamins E) (27, 30, 31).

The calculation of the dietary antioxidant index was estimated by subtracting the average value of the participant's nutrient intake and dividing the result by the standard deviation of the participants (32), and the calculation formula is as follows:


$$\begin{array}{l} \text{CDAI}=\overset{\text{n }=\;6}{\underset{\text{i }=\;1}{\sum }}\frac{\text{Individual Intake }-\text{Mean}}{\text{SD}}\; \end{array}$$

### 2.3 Anthropometric measurements

Anthropometric measurements are widely regarded as a rapid screening method for assessing the nutritional status of children (33). Anthropometric measurement's indicator of this study, including height and weight, adopted standardized methods for each study measurements (34). Participants were asked to remove their shoes, hat, and outer clothing, maintain an upright posture, look straight ahead with both eyes level for the height measurement. When measuring weight, it was also required to take off shoes, hat, and outer clothing, maintain an upright posture, and place the weight scale on a flat surface. Measurements were conducted by trained investigators. After that, children's body mass index (BMI) was calculated. And the BMI percentile was used to classify study participants into underweight/normal weight (<85th percentile), overweight (85 ≤ BMI <95th percentile), and obese (≥95th percentile) in this study (35, 36).

### 2.4 Study covariates

Based on previous studies, we adjusted some covariates to analyze the relationship between CDAI and children growth indicators (37). We included the following factors as covariates in the analysis: age (in years), gender (categorized as male and female), ethnicity (including Han, Hui, Manchu, and other minority nationalities), and educational attainment (nursery education, primary school, senior middle school, junior college, bachelor degree above) in CCSMDM. In addition, these covariates were also included in NHANES, except for different racial classifications (classification as Mexican American, Other Hispanic, Non-Hispanic White, Non-Hispanic Black, Non-Hispanic Asian and Other Race—Including Multi-Racial). BMI was assessed as weight in kilograms divided by height in meters squared. Besides, height, weight, energy, vitamin A, vitamin C, vitamin E, magnesium, zinc, and selenium intake situation were measured at baseline.

### 2.5 Data analysis

In the data analysis process for this study, considering the complexity of the sampling method, a complex weighted statistical analysis of the study population was carried out in NHANES. We conducted all participants descriptive analyses of demographic characteristics, applying mean ± standard deviation for continuous variables and frequency (percentage) for categorical variables.

This research adopted the multinomial logistics regression, three models were built to study the relationship between the CDAI and growth indicators. Model I was a crude model. Model II adjusted for age and gender. Model III added control variables such as the race/ethnic, educational level of children education, level of parental education on the basis of Model II. After adjusting for covariates, the subgroup analyses were performed according to gender using logistic regression analysis. All statistical analyses were conducted R language (version 4.3.1), and two-tailed *p* < 0.05 were considered statistically significant.

## 3 Results

### 3.1 Characteristics of the study population

Table 1 shows the baseline characteristics of all participants in two cohorts, including age, gender, race, growth indicators (BMI, height, and weight), energy, antioxidant nutrients (vitamin A, C, and E, zinc, magnesium, and selenium). Among the 1,064 participants in CCSMDM, the mean age was 7.4 ± 2.7 years, and the mean energy intake was 1421.1 ± 519.9 kcal, with 50.4% being male. In contrast, among the 2,404 participants in NHANES, the mean age was 8.98 ± 2.02 years, and the mean energy intake was 1869.5 ± 550.4 kcal, with males accounting for 50.9%.

**Table 1:** Characteristics of study population from CCSMDM and NHANES.

**Characteristics**	**CCSMDM**			**NHANES**		
	**Overall (*N* = 1,064)**	**Male (*N* = 536)**	**Female (*N* = 528)**	**Overall (*N* = 2,404)**	**Male (*N* = 1,223)**	**Female (*N* = 1,181)**
Age (years), mean (SD)	7.4 (2.7)	7.3 (2.7)	7.5 (2.7)	8.98 (2.02)	8.9 (2.0)	9.1 (2.0)
**Gender, n (%)**						
Male	536 (50.4)			1,223 (50.9)		
Female	528 (49.6)			1,181 (49.1)		
**Nation, n (%)**						
Han group	1,044 (98.1)	529 (98.7)	515 (97.5)			
Hui group	4 (0.4)		4 (0.8)			
Manchu	6 (0.6)	3 (0.6)	3 (0.6)			
other	10 (0.9)	4 (0.7)	6 (1.1)			
**Race, n (%)**						
Mexican American				604 (25.1)	292 (23.9)	312 (26.4)
Other Hispanic				262 (10.9)	154 (12.6)	108 (9.1)
Non-Hispanic White				780 (32.4)	413 (33.8)	367 (31.1)
Non-Hispanic Black				339 (14.1)	159 (13.0)	180 (15.2)
Non-Hispanic Asian				282 (11.7)	140 (11.4)	142 (12.0)
Other Race				137 (5.7)	65 (5.3)	72 (6.1)
Height (cm), mean (SD)	125.4 (18.2)	125.0 (18.3)	125.9 (18.2)	137.6 (14.1)	137.2 (13.9)	137.9 (14.3)
Weight (kg), mean (SD)	28.5 (12.0)	29.0 (12.6)	28.1 (11.3)	37.1 (14.3)	36.4 (13.7)	37.8 (14.9)
BMI (kg/m^2^), mean (SD)	17.4 (3.4)	17.6 (3.3)	17.1 (3.5)	18.9 (4.3)	18.7 (4.1)	19.2 (4.6)
Energy (kcal), mean (SD)	1421.1 (519.9)	1442.0 (524.0)	1400.0 (515.2)	1869.5 (550.4)	1955.0 (562.1)	1781.1 (523.7)
**CDAI, mean (SD)**						
Vitamin A (ug), mean (SD)	494.0 (314.4)	504.7 (320.2)	483.2 (308.3)	632.9 (372.2)	672.1 (419.5)	592.3 (310.9)
Vitamin E (mg), mean (SD)	13.4 (8.6)	13.4 (8.7)	13.4 (8.5)	7.0 (3.3)	7.2 (3.4)	6.8 (3.1)
Vitamin C (mg), mean (SD)	69.6 (67.3)	69.4 (66.6)	69.9 (68.0)	77.0 (56.6)	79.6 (61.0)	74.2 (51.6)
Manganese (mg), mean (SD)	216.5 (96.0)	217.3 (94.2)	215.7 (97.9)	238.3 (79.1)	248.6 (83.4)	227.6 (72.9)
Selenium (ug), mean (SD)	38.8 (22.5)	40.1 (22.8)	37.3 (22.1)	96.9 (37.6)	101.5 (36.8)	92.1 (37.7)
Zinc (mg), mean (SD)	8.2 (3.8)	8.26 (3.8)	8.1 (3.9)	10.0 (4.0)	10.5 (4.1)	9.6 (3.9)

### 3.2 Association between CDAI and children growth indicators

We evaluated the relationship between CDAI and children growth indicators by applying multinomial logistics regression analyses. Table 2 showed the results of logistic regression surveyed by NHANES and CCSMDM, three models were constructed to examine the relationship between CDAI and children growth indicators (BMI, height, weight) in this study. In the crude model, the children growth indicators (BMI, height, weight), the OR of height were 1.31 (95% CI: 1.17~1.50) and 1.11 (95% CI: 1.06~1.17), respectively. The OR of weight were 1.36 (95% CI: 1.23~1.52) and 1.13 (95% CI: 1.07~1.19), respectively. In the fully adjusted model (model 3), the CDAI was positively associated with height (CCSMDM: OR = 1.21, 95% CI: 1.04~1.43, *p* = 0.017; NHANES: OR = 1.11, 95%CI: 1.04~1.18, *p* = 0.001), and weight (CCSMDM: OR = 1.27, 95% CI: 1.09~1.52, *p* = 0.004; NHANES: OR = 1.12, 95%CI: 1.05~1.19, *p* < 0.001). But there was no significant association between CDAI and BMI in children.

**Table 2:** Multinomial logistics regression models of the association between composite dietary antioxidant index and children growth indicators in CCSMDM and NHANES.

	**Model 1**		**Model 2**		**Model 3**	
	**OR [95% CI]**	***P*-value**	**OR [95% CI]**	***P*-value**	**OR [95% CI]**	***P*-value**
**CCSMDAM**						
Height	1.31 [1.17, 1.50]	< 0.001	1.17 [1.03, 1.37]	0.025	1.21 [1.04, 1.43]	0.017
Weight	1.36 [1.23, 1.52]	< 0.001	1.24 [1.08, 1.46]	0.005	1.27 [1.09, 1.52]	0.004
BMI	1.02 [0.96, 1.10]	0.485	0.98 [0.92, 1.06]	0.598	0.98 [0.91, 1.06]	0.554
**NHANES**						
Height	1.11 [1.06, 1.17]	< 0.001	1.11 [1.04, 1.18]	< 0.001	1.11 [1.04, 1.18]	0.001
Weight	1.13 [1.07, 1.19]	< 0.001	1.13 [1.06, 1.20]	< 0.001	1.12 [1.05, 1.19]	< 0.001
BMI	1.05 [1.00, 1.10]	0.039	1.04 [0.99, 1.10]	0.108	1.05 [1.00, 1.11]	0.054

^*^Model 1: Unadjusted model. Model 2: Adjusted for age and gender. Model 3: (CCSMDM) Additionally adjusted for nation, children education, parents' education level. (NHANES) additionally adjusted for race, children education, parents' education level.

### 3.3 Association between CDAI (components) and children growth indicators

To determine the antioxidant nutrients that play a major role in the diet, we analyzed whether there was a correlation between various antioxidant nutrients and children growth indicators in CCSMDM and NHANES database.

Table 3 showed the relationship between antioxidant nutrients and children growth indicators in two cohorts. In the unadjusted covariates model, dietary antioxidants besides vitamin C were correlated with children growth indicators (height and weight; *p* < 0.05) in surveyed by NHANES and CCSMDM. After adjusted covariates (model 3), in CCSMDM, the intakes of vitamins A, C and magnesium significantly correlated between the six components of CDAI and height. And in NHANES, the dietary antioxidants besides vitamin C were correlated with children height. These results indicated that antioxidant nutrients closely related to height were vitamins A, C and magnesium, and the antioxidant nutrients significantly correlated to weight were vitamin A, vitamin E, zinc, selenium and magnesium.

**Table 3:** The correlation between composite dietary antioxidant index (Components) and children growth indicators in CCSMDM and NHANES.

	**Model 1**		**Model 2**		**Model 3**	
	**OR [95% CI]**	***P*-value**	**OR [95% CI]**	***P*-value**	**OR [95% CI]**	***P*-value**
**CCSMDAM**						
**Vitamin A**						
Height	2.36 [1.34, 4.67]	0.008	1.71 [1.03, 3.15]	0.058	1.61 [0.94, 2.98]	0.090
Weight	3.23 [1.91, 5.80]	< 0.001	2.40 [1.31, 4.75]	0.008	2.31 [1.23, 4.76]	0.017
BMI	0.93 [0.74, 1.23]	0.557	0.85 [0.68, 1.12]	0.196	0.84 [0.67, 1.11]	0.176
**Vitamin C**						
Height	17.76 [6.44, 55.49]	< 0.001	21.68 [5.90, 92.76]	< 0.001	21.69 [5.55, 100.60]	< 0.001
Weight	5.98 [2.68, 15.23]	< 0.001	3.36 [1.28, 10.35]	0.024	3.09 [1.19, 9.44]	0.034
BMI	1.04 [0.80, 1.46]	0.797	0.90 [0.69, 1.26]	0.477	0.90 [0.68, 1.27]	0.481
**Vitamin E**						
Height	1.57 [1.13, 2.34]	0.015	0.96 [0.55, 1.73]	0.895	1.66 [0.83, 3.49]	0.166
Weight	1.99 [1.33, 3.21]	0.002	1.54 [0.85, 2.93]	0.172	2.30 [1.16, 4.94]	0.024
BMI	1.17 [0.88, 1.68]	0.333	1.05 [0.76, 1.52]	0.794	1.08 [0.76, 1.62]	0.703
**Zinc**						
Height	4.09 [2.00, 9.69]	< 0.001	1.81 [1.07, 3.35]	0.040	1.87 [1.05, 3.64]	0.044
Weight	4.45 [2.70, 7.85]	< 0.001	1.89 [1.10, 3.62]	0.036	1.97 [1.10, 3.98]	0.039
BMI	1.21 [0.90, 1.69]	0.241	0.98 [0.73, 1.40]	0.899	0.94 [0.68, 1.37]	0.734
**Selenium**						
Height	1.51 [1.15, 2.01]	0.004	1.45 [0.86, 2.59]	0.180	1.23 [0.68, 2.34]	0.519
Weight	1.66 [1.24, 2.25]	0.001	1.95 [1.14, 3.55]	0.021	1.92 [1.08, 3.63]	0.035
BMI	1.04 [0.79, 1.39]	0.797	0.96 [0.72, 1.31]	0.779	0.95 [0.71, 1.32]	0.760
**Manganese**						
Height	2.80 [1.91, 4.27]	< 0.001	1.69 [0.95, 3.16]	0.089	2.20 [1.11, 4.59]	0.029
Weight	3.06 [2.04, 4.76]	< 0.001	1.93 [1.08, 3.64]	0.035	2.25 [1.19, 4.54]	0.017
BMI	1.18 [0.89, 1.65]	0.289	0.99 [0.72, 1.41]	0.931	0.98 [0.70, 1.43]	0.915
**NHANES**						
**Vitamin A**						
Height	1.23 [0.99, 1.55]	0.080	1.35 [1.03, 1.82]	0.036	1.36 [1.02, 1.82]	0.038
Weight	1.30 [1.04, 1.66]	0.031	1.46 [1.10, 1.97]	0.010	1.41 [1.06, 1.90]	0.023
BMI	0.94 [0.81, 1.14]	0.487	0.92 [0.77, 1.13]	0.367	0.92 [0.76, 1.14]	0.402
**Vitamin C**						
Height	1.14 [0.94, 1.40]	0.210	1.53 [1.20, 1.99]	0.001	1.52 [1.19, 1.99]	0.001
Weight	1.06 [0.89, 1.31]	0.511	1.34 [1.07, 1.72]	0.016	1.32 [1.04, 1.69]	0.027
BMI	1.14 [0.94, 1.42]	0.212	1.23 [0.99, 1.55]	0.075	1.23 [0.99, 1.55]	0.073
**Vitamin E**						
Height	1.31 [1.06, 1.64]	0.016	1.15 [0.92, 1.48]	0.234	1.15 [0.92, 1.47]	0.247
Weight	1.46 [1.17, 1.87]	0.001	1.33 [1.04, 1.73]	0.028	1.35 [1.06, 1.78]	0.022
BMI	1.07 [0.88, 1.31]	0.518	1.02 [0.84, 1.26]	0.846	1.11 [0.90, 1.40]	0.347
**Zinc**						
Height	1.63 [1.30, 2.08]	< 0.001	1.48 [1.16, 1.93]	0.002	1.51 [1.18, 1.98]	0.002
Weight	1.61 [1.28, 2.05]	< 0.001	1.45 [1.14, 1.89]	0.004	1.42 [1.11, 1.85]	0.008
BMI	1.31 [1.06, 1.64]	0.014	1.23 [0.99, 1.55]	0.070	1.18 [0.95, 1.50]	0.150
**Selenium**						
Height	1.68 [1.32, 2.15]	< 0.001	1.30 [1.00, 1.71]	0.061	1.33 [1.02, 1.76]	0.042
Weight	1.94 [1.52, 2.51]	< 0.001	1.59 [1.20, 2.14]	0.001	1.59 [1.20, 2.14]	0.001
BMI	1.63 [1.29, 2.08]	< 0.001	1.48 [1.16, 1.92]	0.002	1.57 [1.21, 2.07]	0.001
**Manganese**						
Height	1.54 [1.24, 1.92]	< 0.001	1.44 [1.12, 1.89]	0.006	1.44 [1.12, 1.88]	0.006
Weight	1.60 [1.29, 2.01]	< 0.001	1.53 [1.19, 2.01]	0.001	1.52 [1.18, 1.99]	0.002
BMI	1.17 [0.96, 1.44]	0.129	1.11 [0.90, 1.38]	0.325	1.17 [0.94, 1.47]	0.164

^*^ Model 1: Unadjusted model. Model 2: Adjusted for age and gender. Model 3: (CCSMDM) Additionally adjusted for nation, children education, parents' education level. (NHANES) Additionally adjusted for race, children education, parents' education level.

### 3.4 Subgroup analysis

In addition, to investigate the consistency of the relationship between CDAI and growth indicators across subgroups of different ages and genders, we also conducted a subgroup analysis stratified by gender and age (Table 4). The interaction analysis (all P for interaction >0.05) revealed no statistically significant association in the gender and age subgroup (Supplementary Table 2).

**Table 4:** Subgroup analysis of the association between composite dietary antioxidant index and children growth indicators in CCSMDM and NHANES.

**Subgroup**		**Model 1**		**Model 2**		**Model 3**	
		**OR [95% CI]**	***P*-value**	**OR [95% CI]**	***P*-value**	**OR [95% CI]**	***P*-value**
**Gender**							
Boys	CCSMDAM						
	Height	1.24 [1.08, 1.26]	0.010	1.13 [0.97, 1.37]	0.166	1.16 [0.98, 1.43]	0.108
	Weight	1.33 [1.16, 1.57]	< 0.001	1.23 [1.02, 1.55]	0.054	1.26 [1.04, 1.61]	0.038
	BMI	0.97 [0.89, 1.08]	0.533	0.97 [0.88, 1.08]	0.510	0.97 [0.87, 1.09]	0.561
	NHANES						
	Height	1.16 [1.08, 1.26]	< 0.001	1.17 [1.08, 1.29]	< 0.001	1.17 [1.08, 1.29]	< 0.001
	Weight	1.16 [1.08, 1.25]	< 0.001	1.16 [1.07, 1.28]	0.001	1.16 [1.07, 1.28]	0.001
	BMI	1.06 [0.99, 1.14]	0.133	1.05 [0.98, 1.13]	0.193	1.06 [0.99, 1.15]	0.119
Girls	CCSMDAM						
	Height	1.38 [1.21, 1.60]	< 0.001	1.24 [1.00, 1.57]	0.058	1.29 [1.00, 1.72]	0.067
	Weight	1.39 [1.21, 1.62]	< 0.001	1.25 [1.01, 1.58]	0.048	1.30 [1.02, 1.71]	0.047
	BMI	1.06 [0.97, 1.17]	0.205	0.99 [0.90, 1.10]	0.851	0.98 [0.89, 1.10]	0.763
	NHANES						
	Height	1.06 [0.99, 1.15]	0.117	1.04 [0.96, 1.14]	0.326	1.05 [0.97, 1.15]	0.237
	Weight	1.10 [1.02, 1.19]	0.020	1.09 [1.00, 1.19]	0.066	1.08 [0.99, 1.18]	0.091
	BMI	1.04 [0.97, 1.12]	0.245	1.04 [0.97, 1.11]	0.325	1.05 [0.97, 1.13]	0.226
**Age (year)**							
≤ 6 years old	CCSMDAM						
	Height	1.17 [1.04, 1.35]	0.017	1.17 [1.03, 1.37]	0.025	1.21 [1.04, 1.43]	0.017
	Weight	1.25 [1.09, 1.44]	0.002	1.24 [1.08, 1.46]	0.006	1.27 [1.09, 1.52]	0.004
	BMI	1.04 [0.92, 1.20]	0.604	1.03 [0.90, 1.19]	0.733	1.05 [0.91, 1.24]	0.544
	NHANES						
	Height	1.13 [1.06, 1.21]	0.001	1.14 [1.06, 1.23]	< 0.001	1.14 [1.06, 1.22]	< 0.001
	Weight	1.14 [1.06, 1.23]	0.001	1.15 [1.07, 1.24]	< 0.001	1.14 [1.06, 1.23]	0.001
	BMI	1.05 [0.96, 1.17]	0.333	1.05 [0.95, 1.16]	0.382	1.04 [0.94, 1.16]	0.462
>6 years old	CCSMDAM						
	Height	0.99 [0.96, 1.03]	0.727	1.00 [0.96, 1.05]	0.859	0.99 [0.95, 1.04]	0.752
	Weight	0.97 [0.93, 1.00]	0.083	0.97 [0.93, 1.01]	0.100	0.96 [0.92, 1.00]	0.094
	BMI	0.95 [0.87, 1.04]	0.253	0.95 [0.87, 1.04]	0.264	0.95 [0.87, 1.05]	0.296
	NHANES						
	Height	1.06 [0.99, 1.15]	0.711	1.00 [0.89, 1.13]	0.939	1.00 [0.89, 1.14]	0.972
	Weight	1.10 [1.02, 1.19]	0.144	1.06 [0.96, 1.19]	0.285	1.07 [0.96, 1.20]	0.228
	BMI	1.04 [0.99, 1.11]	0.119	1.04 [0.99, 1.11]	0.149	1.06 [1.00, 1.13]	0.078

^*^ Model 1: Unadjusted model. Model 2: Adjusted for age and gender. Model 3: (CCSMDM) Additionally adjusted for nation, children education, parents' education level. (NHANES) Additionally adjusted for race, children education, parents' education level.

After adjusted for covariates (model 3), CDAI was significantly correlated with height and weight in boys in two cohorts; in girls, CDAI was significantly positively correlated with weight. In addition, CDAI was significantly correlated with height and weight in children ≤ 6 years of age in both two cohorts, whereas this association became non-significant in children >6 years of age.

**Figure 2:**
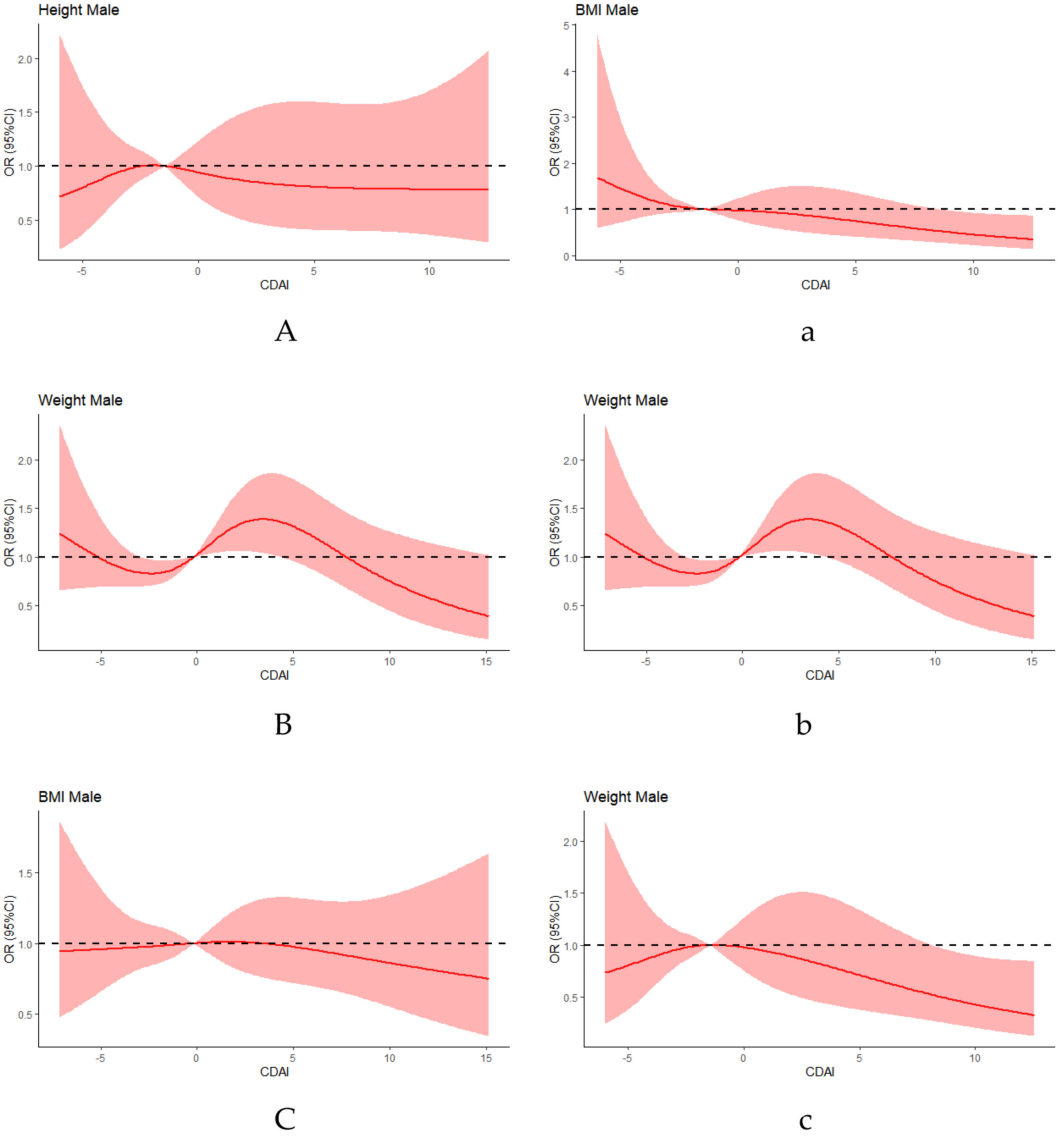
Association of male CDAI with children growth indicators in a resticted cubic spline among all participants. **(A, B, C)** CCSMDM was adjusted for age, gender, nation, education level and parents' education level. **(a, b, c)** NHANES was adjusted for age, gender, race, education level and parents' education level.

Furthermore, multivariate adjusted RCS analysis showed a non-linear relationship between CDAI and various antioxidant nutrients (vitamin C, zinc; CCSMDM: *p* < 0.001; NHANES: *p* < 0.001; Figure 2).

## 4 Discussion

This cross-sectional study employed the CCSMDM and NHANES databases to examine the relationship between CDAI and growth indicators in children aged 3–12 years. Preliminary results suggested a significant correlation between CDAI and children growth indicators. The relationship persisted even after adjusting for all covariates, suggesting that the dietary intake of antioxidant nutrients may serve as a protective factor for growth and development. Reasonable dietary and increased antioxidant intake can help prevent dysplasia and short stature (38).

Currently, growth and development in children have been recognized as major topics of global attention, and delayed growth and development in children may be related to malnutrition (39). Nowadays, unhealthy eating habits, such as consuming foods high in calories, fat, and sugar, can lead to deficiencies in essential nutrients, potentially impairing growth and development (40, 41). Many studies have investigated the association between single antioxidant nutrient and the growth and development of children (42–44). Micronutrient deficiencies may affect children's linear growth in cross-sectional studies of Indian girls (20). A cross-sectional study conducted by Hamam Hadi et al. (45) found that high-dose vitamin A supplementation improved the linear growth of children with very low serum retinol. Besides, some studies suggested that growth stunting was associated with marginal deficiencies of several micronutrients, and supplementation with single nutrient will not have a significant for linear growth (46, 47). The types of nutrients we consume in our daily lives are quite diverse, and a single nutrient does not suggest dietary total antioxidant capacity (48). Therefore, we introduced a comprehensive scoring system CDAI to assess the total dietary antioxidant levels. The CDAI can reflect an individual's antioxidant profile, that was closer to real life, without the interaction of various antioxidants. This cross-sectional study based on two cohorts found the association between CDAI and growth and development in children, after adjusted covariates.

Moreover, this study showed intake of the dietary antioxidants vitamins A, C, and magnesium was associated with height, and the antioxidants significantly correlated to weight were vitamin A, vitamin E, zinc, selenium, and magnesium. The micronutrients we consume in our daily lives mainly include vitamins and minerals, which come from diverse sources such as meat, plant-based foods, drinking water, and other mineral-rich sources (49). Given the complexity of people's daily dietary intake, achieving beneficial effects on growth and development by simply increasing the intake of a single food item may be challenging. A more effective approach likely involves the combined consumption of a variety of foods. Rivera et al. (50) found that lack of micronutrients can lead to growth retardation in children. The most prevalent micronutrient deficiencies are vitamin A, zinc, vitamin E, etc. Vitamin A deficiency primarily affects children in impoverished regions, while vitamin E deficiency is commonly observed in developing countries due to dietary fat absorption irregularities. Zinc deficiency can result in growth inhibition, puberty issues, inflammation, and gastrointestinal or skin problems (48, 51–53). Notably, dietary habits may influence antioxidant nutrient intake. Our results showed significant differences in the odds ratio (OR) values for vitamin C between the two datasets, reflecting variations in dietary intake across different populations. For example, Chinese dietary habits typically include a higher intake of vegetables, which results in relatively greater vitamin C consumption (54, 55). In contrast, Americans tend to consume more meat, leading to a comparatively lower intake of vitamin C (56). The sources of vitamin A also differ between populations due to differences in dietary habits. Much of the vitamin A consumed comes from plant-based foods in China, particularly brightly colored vegetables. In contrast, in the United States, vitamin A is more commonly obtained from animal-based sources, such as liver, dairy products, and eggs, which provide retinol (57). In addition to lifestyle patterns between the two cohorts, potential biases in the results may also be attributed to cultural, environmental, and genetic variations, as well as inconsistencies in dietary assessment methodologies. Although the sources of micronutrients may differ, both datasets consistently show that the intake of antioxidant micronutrients in the diet is beneficial for children's growth and development, further reinforcing the reliability of our findings.

Dietary antioxidants are known to have an intervention effect in adverse health effects such as OS and chronic inflammation, but the association between CDAI and the growth and development is currently unknown. Existing evidence suggests unreasonable dietary patterns or Hyper-Caloric food intake, which may lead to an increase in reactive oxygen species levels, and further triggering chronic inflammatory response (58, 59). Żegleń et al. (60) analyzed the total antioxidant plasma capacity in pediatric patients with obesity and found that it was lower than the adult population with obesity. As a result, children may be more susceptible to OS than adults due to an incomplete development of the antioxidant system (61). To the best of our knowledge, consuming antioxidants or antioxidant nutrients could help slow down the occurrence of the OS (62). This study indicated that there was a significant correlation between antioxidant nutrients (vitamin C, zinc (16), and magnesium) and height. In addition, antioxidant nutrients were significantly correlated with body weight. These results were consistent with previous studies (63).

## 5 Strengths and limitations of the study

This research indicates that children's growth and development abilities are influenced by CDAI, and a moderate intake of antioxidants was beneficial for the growth and development of children. Our study has several noteworthy strengths. First, this research holds significance as the first to investigate the relationship between dietary antioxidant nutrients and growth and development status in children. Second, our study considers the CDAI, which has superior application advantages and validity to the traditional dietary antioxidant (64). Furthermore, the study used two databases to explore the relationship between CDAI and children growth indicators. Finally, this analysis provides intervention directions for promoting children growth indicators. Of course, there are still some limitations in this study. Firstly, this study did not account for potential confounding factors including macronutrient intake (proteins, fats, and carbohydrates), dietary quality assessments, or biochemical blood parameters, which may influence the interpretation of antioxidant-growth correlations. Secondly, the mechanism by which antioxidant nutrients affect the growth and development of children is still unclear. It is therefore necessary to carry out further research on the relevant physiological mechanisms in the future. Thirdly, NHANES dietary assessments employ two sequential 24-h recall interviews, with potential measurement errors constituting unexplained variance in our analytical framework. Finally, we cannot make any causal inferences due to the cross-sectional design of the study.

## 6 Conclusions

In summary, our findings indicated that a higher level of dietary antioxidant nutrients may help to promote children growth indicators. This result highlighted the importance of intake of antioxidant-rich foods for promoting children growth indicators. Our study is the first to investigate the relationship between dietary antioxidant nutrients and the growth and development status of children. We used two databases for validation to enhance reliability of the results. These findings support the adoption of balanced diets and encourage the consumption of more dietary antioxidants to promote children's growth indicators. However, we cannot make any causal inferences due to the cross-sectional design of the study. Further validation is needed through large prospective cohort study or randomized controlled trials.

## Data Availability

The datasets presented in this study can be found in online repositories. The names of the repository/repositories and accession number(s) can be found at: https://wwwn.cdc.gov/nchs/nhanes/default.aspx.
