# Supplementary material for: Relationship between composite dietary antioxidants index and growth indicators in children aged 3–12 years: results from two observational studies
Source: Front Nutr. 2025 Mar 20;12:1551754. doi: 10.3389/fnut.2025.1551754 (PMC11965125; doi:10.3389/fnut.2025.1551754)
Supplement: Supplementary file 1 [file Table_1.docx]

Supplementary Table 1 Basic characteristics of two queues before and after energy adjustment

|  | CCSMDAM | NHANES |  | CCSMDAM | NHANES |  |
| --- | --- | --- | --- | --- | --- | --- |
|  | Unadjusted energy | Unadjusted energy | *P*-value | Adjusted energy | Adjusted energy | *P*-value |
| Vitamin A (ug),  mean (SD) | 494.0 (314.4) | 632.9 (372.2) | ＜0.001 | 362.15 (195.12) | 343.33 (183.62) | 0.006 |
| Vitamin C (mg),  mean (SD) | 69.6 (67.3) | 77.0 (56.6) | ＜0.001 | 47.59 (36.16) | 42.10 (29.71) | ＜0.001 |
| Vitamin E (mg),  mean (SD) | 13.4 (8.6) | 7.0 (3.3) | ＜0.001 | 9.32 (3.73) | 3.76 (1.46) | ＜0.001 |
| Zinc (mg),  mean (SD) | 8.2 (3.8) | 10.0 (4.0) | ＜0.001 | 5.76 (1.67) | 5.44 (1.68) | ＜0.001 |
| Selenium (ug), mean (SD) | 38.8 (22.5) | 96.9 (37.6) | ＜0.001 | 27.32 (12.41) | 52.24 (14.41) | ＜0.001 |
| Manganese (mg), mean (SD) | 216.5 (96.0) | 238.3 (79.1) | ＜0.001 | 152.88 (37.53) | 129.02 (27.98) | ＜0.001 |

Supplementary Table 2 Gender and age interaction of the association between

CDAI and growth indicators

| Subgroup |  | *p* for interaction |
| --- | --- | --- |
| Gender | CCSMDAM |  |
|  | Height | 0.437 |
|  | Weight | 0.208 |
|  | BMI | 0.312 |
|  | NHANES |  |
|  | Height | 0.065 |
|  | Weight | 0.205 |
|  | BMI | 0.824 |
| Age (year) | CCSMDAM |  |
|  | Height | 0.373 |
|  | Weight | 0.896 |
|  | BMI | 0.608 |
|  | NHANES |  |
|  | Height | 0.138 |
|  | Weight | 0.378 |
|  | BMI | 0.879 |
